# Shaping Leg Muscles in *Drosophila*: Role of *ladybird,* a Conserved Regulator of Appendicular Myogenesis

**DOI:** 10.1371/journal.pone.0000122

**Published:** 2006-12-27

**Authors:** Tariq Maqbool, Cedric Soler, Teresa Jagla, Malgorzata Daczewska, Neha Lodha, Sudhir Palliyil, K. VijayRaghavan, Krzysztof Jagla

**Affiliations:** 1 Institut National de la Santé et de la Recherche Médicale U384, Faculté de Medecine, Clermont-Ferrand, France; 2 National Centre for Biological Sciences, Tata Institute of Fundamental Research, Bangalore, India; 3 Department of General Zoology, Wroclaw University, Wroclaw, Poland; Baylor College of Medicine, United States of America

## Abstract

Legs are locomotor appendages used by a variety of evolutionarily distant vertebrates and invertebrates. The primary biological leg function, locomotion, requires the formation of a specialised appendicular musculature. Here we report evidence that *ladybird*, an orthologue of the *Lbx1* gene recognised as a hallmark of appendicular myogenesis in vertebrates, is expressed in leg myoblasts, and regulates the shape, ultrastructure and functional properties of leg muscles in *Drosophila*. *ladybird* expression is progressively activated in myoblasts associated with the imaginal leg disc and precedes that of the founder cell marker *dumbfounded*. The RNAi-mediated attenuation of *ladybird* expression alters properties of developing myotubes, impairing their ability to grow and interact with the internal tendons and epithelial attachment sites. It also affects sarcomeric ultrastructure, resulting in reduced leg muscle performance and impaired mobility in surviving flies. The over-expression of *ladybird* also results in an abnormal pattern of dorsally located leg muscles, indicating different requirements for *ladybird* in dorsal versus ventral muscles. This differential effect is consistent with the higher level of Ladybird in ventrally located myoblasts and with positive *ladybird* regulation by extrinsic Wingless signalling from the ventral epithelium. In addition, *ladybird* expression correlates with that of FGF receptor Heartless and the read-out of FGF signalling downstream of FGF. FGF signals regulate the number of leg disc associated myoblasts and are able to accelerate myogenic differentiation by activating *ladybird*, leading to ectopic muscle fibre formation. A key role for *ladybird* in leg myogenesis is further supported by its capacity to repress *vestigial* and to down-regulate the *vestigial*-governed flight muscle developmental programme. Thus in *Drosophila* like in vertebrates, appendicular muscles develop from a specialised pool of myoblasts expressing *ladybird/Lbx1*. The *ladybird/Lbx1* gene family appears as a part of an ancient genetic circuitry determining leg-specific properties of myoblasts and making an appendage adapted for locomotion.

## Introduction

Skeletal leg musculature is required for walking in all animals, but the genetic mechanisms that control its development have been analysed mainly in vertebrates [1]–[6]. Although much knowledge has been gained from these studies, little is known about the mechanisms governing patterning and diversification of leg muscles, pointing to a need for other model systems to study these processes. Interestingly, the conserved family of *Distal-less/Dlx* homeobox genes was found to be involved in outgrowth of appendages over a broad spectrum of proteostome and deuterostome phyla, suggesting the existence of ancient genetic circuitry controlling leg development [7]–[9]. This prompted us to find out whether the genetic programme governing leg muscle formation, required for the main biological leg function, which is locomotion, was under the control of conserved genes. With this aim we investigated myogenic functions of genes known to control vertebrate appendicular myogenesis in *Drosophila*.

In *Drosophila* leg muscles derive from myoblasts associated with the leg imaginal disc. The leg disc is a flat epithelial sheet of cells during the first and second larval instar stages. With the onset of third instar, though still monolayered, it begins to develop concentric folds, undergoes cell shape changes, and divides into the leg disc proper and a proximal region that corresponds to the ventral thorax/adult body wall. It has been shown that these two regions have different genetic requirements [10], [11]. During the pupal stage, the disc epithelium telescopes out from its centre and elongates along the proximal-distal axis to make the slender adult leg epidermis by cell rearrangement [12], [13]. The signalling pathways and factors that control patterning of leg disc epithelium have been extensively studied [14]–[18]. However, the mechanisms governing the myogenic programme in the developing leg disc remain largely unknown.

Previous studies indicate that two different developmental strategies are used during the development of adult *Drosophila* muscles, namely muscle template-based myogenesis and *de novo* muscle formation. A subset of indirect flight muscles IFMs, the dorsal longitudinal muscles DLMs, uses larval templates for formation [19], [20], whereas the other set of IFMs, the dorsoventral muscles DVMs, the direct flight muscles DFMs and leg muscles develop *de novo* from a pool of *twist*-positive precursors of adult muscles [21], [22]. These pathways imply distinct regulatory mechanisms that control the development of sets of flight muscles and the muscles of the leg. It has been demonstrated [23]–[25] that the homeobox genes *cut* and *apterous* *ap* are autonomously required for the formation of DFMs, whereas *vestigial vg* controls the formation of IFMs. Besides these intrinsic factors, Wingless Wg signalling from the imaginal disc epithelium contributes to the functional diversification of myoblasts forming DLMs and DFMs [25]. The myogenic role of extrinsic Wg is reminiscent of that of its vertebrate counterpart Wnt6 expressed in the ectoderm overlying dorsal somites [26], [27] and involved in the specification of myogenic progenitors in the dermomyotome. Interestingly, the initially distinct genetic pathways underlying template-based and *de novo* adult muscle formation in *Drosophila* converge to activate the muscle founder cell marker, *dumbfounded* *duf* [22], [28]. During embryonic myogenesis, segregation of *duf*-positive muscle founders is mediated by the Notch pathway [29]–[31]. They express a combinatorial code of transcription factors, known as muscle identity genes, and are thought to carry all the information required to display unique properties of resulting muscle fibres [32]. As demonstrated recently by Dutta et al., [33] in adult thoracic and abdominal myogenesis, Duf is initially activated in all myoblasts and subsequently founder myoblasts are chosen by Htl-transduced FGF signals by the up-regulation of *Duf* in a subset of myoblasts corresponding to differentiating founders. *Duf* is down-regulated in other myoblasts that will become fusion competent cells. Thus adult *Drosophila* myogenesis also involves founder myoblast selection through FGF-dependent signalling. In chick embryos, the FGF8 and its receptor FREK/FGFR4 were found to promote muscle differentiation [34], indicating potentially convergent roles for FGF in invertebrate and vertebrate myogenic programs. In addition, several transcription factors have been found to control somitic and appendicular myogenesis in vertebrates [5], [27]. One of these genes is the *Lbx1,* specifically expressed in appendicular myoblasts and required for their migration into the limb buds [2], [3], [35].

Here we show that the invertebrate counterpart of *Lbx1*, *ladybird lb*, known to specify the identity of a subset of embryonic *Drosophila* muscles [36], plays a key role in adult leg myogenesis in *Drosophila*. The leg disc associated *lb* expression is positively regulated by extrinsic Wg signals and is a key marker for the appendicular myogenic programme. *lb* expression precedes that of *duf* and coincides with the read-out of FGF signalling DOF. Our data demonstrate that during the development of adult *Drosophila* muscles *lb* is required for the establishment of morphological, ultrastructural and functional properties of leg muscles. Thus it is likely that the *ladybird/Lbx1* gene family is part of an ancient genetic circuitry that makes an appendage adapted for locomotion through the control of appendicular muscle development.

## Results

### *ladybird early* is expressed in leg disc associated myoblasts

**Figure 1:**
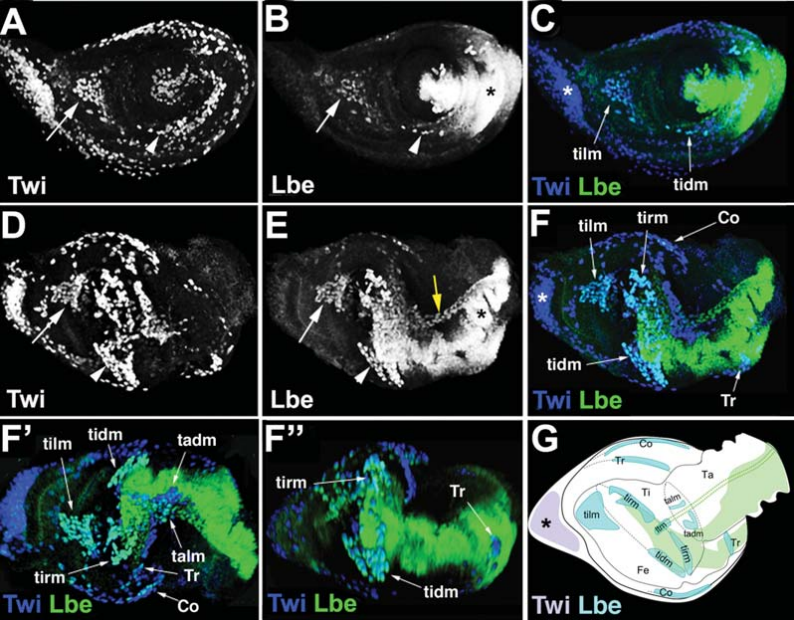
Lbe is dynamically expressed in leg disc myoblasts. A–F Confocal images of leg imaginal discs stained with anti-Twi A, D and blue in merge C, F, and anti-Lbe B, E and green in merge C, F antibodies. A–C Third instar leg imaginal disc. B Lbe expression can be seen in subsets of Twi myoblasts, associated with regions of the leg disc that develop into different segments of the adult leg. Arrows in B, E show a group of myoblasts that give rise to dorsal femur muscle, tilm. The arrowheads in B, E point to precursors of ventral femur muscle, tidm. D–F″ 0 hr APF leg imaginal disc. Myoblasts are regionalised at this stage and are seen at future muscle-forming sites in adult tibia tadm, talm, femur tidm, tilm, trocanter Tr, coxa Co. Lbe is expressed in almost all Twist myoblast subsets at specific sites along the proximal-distal axis in the leg disc proper. Most proximal cells including the dorsal proximal myoblasts asterisks in C, F are devoid of Lbe expression. F′, F″ Two different views of a 3D reconstruction see also Video 1S of the disc presented in F showing spatial distribution of different groups of leg myoblasts. G The schematic of F showing positions of Lbe-positive myoblasts within the leg segments. Lbe is also expressed in leg disc epithelium in the ventral region black asterisks in B, E and the long tendon yellow arrow in E. Abbreviations: Ta, tarsus, Ti, tibia, Fe, femur, see also 22 for muscle nomenclature.

Earlier work [22] showed the dorso-ventral organisation of *Drosophila* leg muscles to be similar to that observed in the vertebrate leg. This suggested that gaining insights into the genetic control of leg muscle patterning in the fruit fly could improve our understanding of how appendicular myogenesis is regulated in general. We found that *ladybird early lbe*, an orthologue of a key regulator of appendicular myogenesis in vertebrates, *Lbx1* [27], is dynamically expressed in myoblasts associated with the leg imaginal disc proper Fig. 1, Video S1 and Video S2. As reported previously [21], [22], see also Fig. 1A all leg myoblasts express a bHLH factor Twist Twi. Initially, at early third larval instar, Lbe is detected in a small subset of Twi-expressing cells located in the dorsal femur arrows in Fig. 1A and B and giving rise to the tibia levator muscle tilm Fig. 1C; see also [22] for leg muscle nomenclature. Most ventrally, Lbe marks individual myoblasts arrowheads in Fig. 1A and B that give rise to the tibia depressor muscle tidm Fig. 1C. The number of leg myoblasts expressing Lbe gradually increases compare corresponding groups of myoblasts indicated by arrows and by arrowheads in Figs 1B and 1E, and at the beginning of pupation all of them express at least a low level of Lbe Fig. 1D–F″. In contrast, myoblasts associated with the proximal portion of the leg discs asterisks in Fig. 1C, F and G remain Lbe-negative. To determine the spatial distribution of Lbe-expressing myoblasts, 3D reconstructions of several early pupa discs were performed and visualised using Volocity^TM^ software. The reconstruction of a 0 h APF disc Fig. 1F is shown in Video S1 and Video S2. The 3D analyses reveal that Lbe-positive myoblasts form spatially restricted groups of cells lying at highly stereotyped positions that can be correlated with defined muscles in the adult leg see schematic, Fig. 1G. This is particularly obvious in the tibia and femur Video S1 and snap shots from the 3D-reconstruction, Fig. 1F′ and F″. In these segments, at 0 h APF the groups of Lbe-expressing myoblasts are seen located dorsally and ventrally at positions representing the levator [talm, tilm] and the depressor tadm, tidm muscles. Interestingly, they lie close to Stripe Sr-expressing tendon precursors Video S2, highlighting possible interactions with invaginating internal tendons [22] and their role in the spatial distribution of myoblasts within the leg segments.

**Figure 2:**
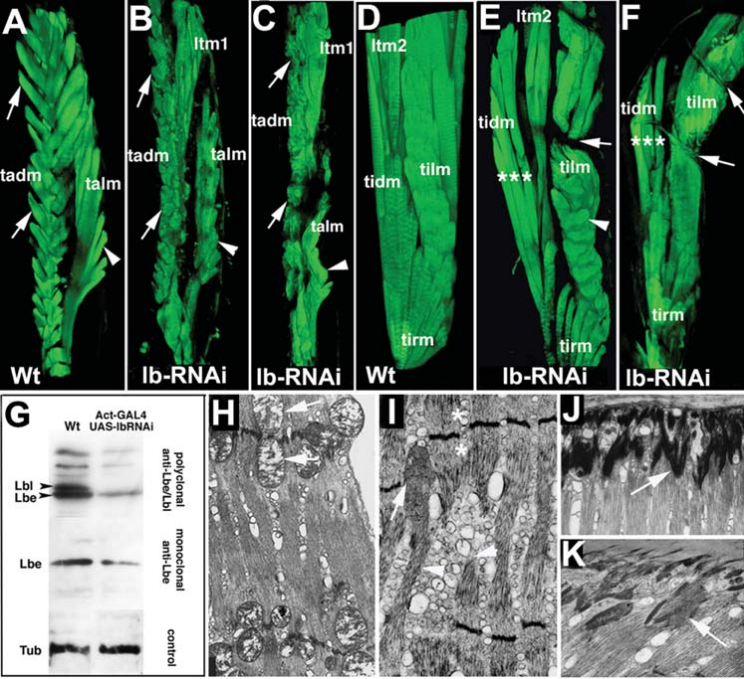
Effects of RNAi-based attenuation of Lb gene function. A–F Anterior views of tibia A–C and femur D–F musculature revealed in wild type A, D and lbRNAi B, C, E, F flies carrying MHC-tauGFP transgene. B, E show mild phenotypes whereas C, F show severe lbRNAi phenotypes. 3D reconstructions from confocal scans were used to generate the presented views see corresponding 3D videos in Video S3-S8. Muscle fibres from *lb*RNAi legs are smaller in both ventral arrows in A–C and dorsal arrowheads in A–C tibia. General muscle mass appears reduced in tibia C and femur asterisks in E and F. Loss of muscle mass and the abnormal attachment of muscle fibres to the leg epithelium lead to morphological defects most frequently manifested by bending of the femur segment arrows in E and F. Note also the altered shape of muscle fibers in tadm muscles B, C and tilm muscle E. G RNAi induced reduction in Lbe and Lbl protein levels revealed by Western blot using two different anti-Lb antibodies. Note that a low level of Lbe is still detected in embryos ubiquitously expressing *lb*RNAi constructs see Materials and Methods for details. H, J Wild type electron microscopy micrographs and I, K micrographs from lbRNAi femur muscles showing H, I sarcomeric ultrastructure and J, K muscle-tendon junction area. The Z line associated pairs of mitochondria dyads, arrows in H are absent in lbRNAi muscle asterisks in I and the few mitochondria still present arrow in I appear to have altered internal structures. Also, myofilaments from lbRNAi sarcomeres are highly disorganised and some of them are disrupted arrowheads in I. A lower intensity electron dense desmosomes are detected in muscle-tendon junctions from lbRNAi legs arrow in K when compared to the wild type arrow in J.

In addition to myoblasts, Lbe was detected in the ventral portion of the leg disc epithelium and in the Sr-expressing precursor cells of the long tendon yellow arrow in Fig. 2E, see also Video S2.

### *ladybird* is required for the proper patterning and ultrastructure of leg muscle fibres

Finding that Lbe marks specifically differentiating leg myoblasts suggested an instructive role in leg myogenesis. To characterise Lbe functions we undertook to knock down its activity by RNAi. As the *lb* locus contains the *lbe* and functionally redundant *ladybird late* *lbl* gene [37]–[39], we generated UAS-RNAi lines carrying constructs against both *lb* genes see Materials and Methods for details. One of the generated lines was able significantly to decrease levels of both Lbe and Lbl proteins Fig. 2G. To attenuate *lb* gene activity in myoblasts, the selected UAS-lbRNAi line was crossed to an adult myoblast driver 1151-GAL4 [40] combined with MHCtauGFP [22]. The MHCtauGFP-revealed muscle pattern clearly showed that the general architecture of leg muscle fibres devoid of *lb* function was severely affected Fig. 2A–F and Video 3–Video S8. We can distinguish two classes of phenotypes: i a mild muscle phenotype Figs 2B, E, Video S4 and Video S7, in which the muscle mass and the number of muscle fibres were only slightly reduced; and ii a strong lbRNAi phenotype Figs. 2C, F, Video S5 and Video S8, in which leg muscles were significantly smaller and were composed of a reduced number of fibres. Importantly, in both classes of phenotypes the *lb*-deficient muscle fibres were misshapen and adopted irregular rounded or spiracle-like forms surrounding internal tendons compare tadm fibres indicated by arrows and talm fibres indicated by arrowheads in Fig. 2A and 2B. This was most probably due to abnormal growth of myotubes and/or their impaired ability to recognise epithelial attachment sites. The reduced number of muscle fibres observed in legs displaying strong RNAi phenotypes can result from the degeneration of some of the misshapen myotubes. The loss of leg muscle mass in *lb*-deficient flies was the cause of morphological leg deformations most frequently seen within the femur segment arrows in Fig. 2E, F. To test whether the reduced muscle mass could result from a smaller number of leg myoblasts, we examined Twi expression in discs from the third instar 1151>lbRNAi larvae. No changes in the number of Twi-expressing cells were observed [data not shown], indicating that *lb* is not involved in myoblast proliferation. We had previously shown that adult myoblast driver 1151-GAL4 was also expressed in internal tendon precursors of the leg disc [22]. To rule out any influence of *lb* attenuation in tendon cells on the observed muscle phenotypes or any influence of *lb* attenuation in myoblast on tendon cell specification, we expressed *lb*RNAi using a tendon specific SrGal4;UASGFP and 1151Gal4;UASGFP strains Materials and Methods. We did not find any effects on tendon specification in Stripe>lbRNAi leg discs data not shown. This rules out tendon-mediated non-autonomous effect of *lb* attenuation on muscle phenotypes. We also found that all tendons were present in 1151>lbRNAi legs Fig. S1 ruling out the possibility of a non-specific effect of *lb* attenuation on tendon specification. However, some of the internal tendons appeared reduced yellow arrows in Fig. S1 in 1151>lbRNAi animals, suggesting that their morphogenesis was affected. This is most probably due to a non-autonomous effect of *lb* knock-down in muscles, leading to impaired muscle-tendon interactions, which are known to be required for final tendon morphogenesis [41].

In addition to morphological defects, RNAi-mediated *lb* knock-down induced alterations in the ultrastructure of leg muscle fibres Fig. 2H–K. As revealed by electron microscopy analysis, the number of mitochondria associated with the sarcomeric Z line was significantly reduced arrows in Fig. 2H and asterisks in Fig. 2I and myofilaments appeared interrupted or irregularly arranged arrowheads in Fig. 2I compared with Fig. 2H. Also, a reduced intensity of electron-dense desmosomes is visible at the junction between muscle fibres and the internal tendons compare Fig. 2K and Fig. 2J. Overall, the observed morphological and ultrastructural alterations suggest that attenuation of *lb* may affect muscle fibre assembly and contractility, leading to reduced leg muscle performance.

**Figure 3:**
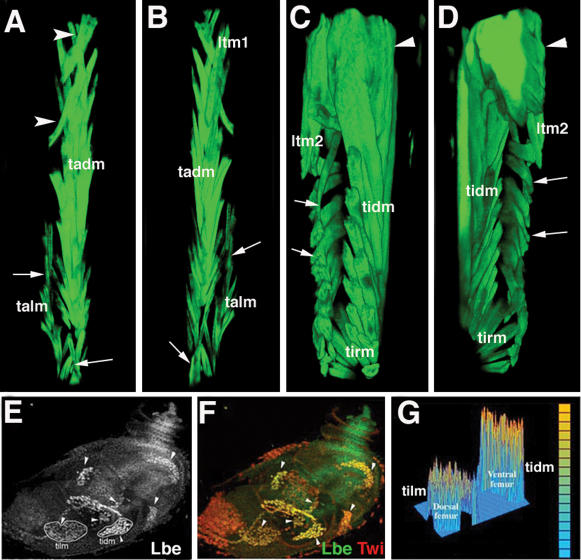
Differential requirement for Lb in dorsal and ventral muscles. A–D Muscle phenotypes observed in A, B tibia and C, D femur after 1151-Gal4 driven overexpression of Lbe. Anterior A, C and posterior B, D views from 3D reconstructed confocal scans. Muscles are visualised by MHC-tauGFP. Reconstructions were performed using Volocity 3.0 software Improvision; see also corresponding 3D Video S9. Arrows in A–D point to abnormally shaped dorsal levator muscles and the tibia reductor muscle. Within the femur C, D dorsal muscle fibres are misoriented and attached on both sides to the epithelium. Arrowheads in A, C, D indicate ectopically located muscle fibres. Within the proximal femur C, D ectopic fibres form a condensed muscle mass. E, F A 0 h APF leg disc showing Lbe expressing groups of myoblasts arrowheads. E Myoblasts that give rise to dorsal tilm and ventral tidm femur muscles are outlined. G Intensity of fluorescence measured within the outlined areas using Fluoview software Olympus. Note that ventrally located femur myoblasts express significantly higher levels of Lbe than those in the dorsal region.

To determine whether increased *lb* expression could also influence leg muscle development, we analysed muscle pattern in 1151-GAL4; MHCtauGFP;UAS-Lbe newborn flies. The 1151-driven gain-of-*lbe* function led to severe defects in the patterning of dorsally located leg muscles Fig. 3A–D. The tibia talm muscle was abnormally shaped arrows in Fig. 3A, B and the homologous levator muscle in the femur [tilm] was completely disrupted arrows in Fig. 3C, D and Video S9. A large proportion of dorsally located femur fibres normally contributing to the tilm muscle were unable to attach to the tilt tendon, most probably leading to its degeneration see Video S9. Instead, they attached on both sides to the disc epithelium, adopting orientations perpendicular to the proximal-distal axis. In addition, some ectopic dorsal muscle fibres were observed in the proximal part of the tibia and femur arrowheads in Fig. 3A–D. Thus when over-expressed *lbe* was able to influence properties of dorsal rather than ventral muscle fibres, suggesting that ventral and dorsal myoblasts may have different requirements for the levels of Lbe protein. To test this possibility we measured intensity of fluorescence of ventral and dorsal femur myoblasts from leg discs stained for Lbe Fig. 3E–G. As shown in Fig. 3E–G, myoblasts that give rise to ventrally located tidm muscle express significantly higher levels of Lbe protein than those growing into dorsal tilm muscle. This observation can explain the sensitivity of dorsal muscles to increased levels of Lbe, indicating that proper patterning of leg muscles depends on precise regulation of *lbe* expression.

As the total number of myoblasts [data not shown] and muscle fibres in Lbe GOF Fig. 3A–D and data not shown and LOF Fig. 2A–F contexts are similar to that in the wild type we conclude that Lbe functions to set properties rather than number of leg myoblasts.

### Role of *ladybird* in leg muscle performance and mobility of adult flies

**Figure 4:**
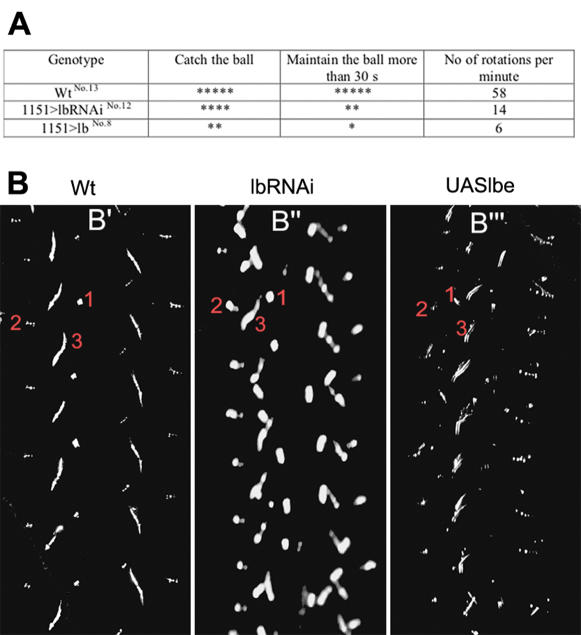
*lb* is required for proper leg muscle performance and walking behaviour A The ball test see Videos S10–S12. The abilities of flies to catch, maintain and rotate a polystyrene ball were tested. The number of individuals tested males only is indicated in upper case after the genotype. Each male performed each of the tests three times. The number of asterisks max. 5 illustrates the average performance. Notice that the RNAi-based attenuation of *lb* leads to a reduced ability to catch about 20% of failures and especially to maintain the ball about 60% of failures with slower and irregular rotations. Defects in catching, maintaining and rotating the ball were comparatively stronger in flies overexpressing *lb*. About 60% of flies were unable to catch the ball and more than 80% lost it in less than 30 s. B The ‘leg-print’ test for walking pattern. Two-day old flies were allowed to walk on a carbon-soot coated glass slide and their tracks were examined. The direction of movement is towards the top of each panel. The imprints made by the first 1 second 2 and third leg 3 of the left hemisegment are marked in each panel. Wild type flies B′ show a stereotypic pattern of prints, a consequence of a ‘tripod’ gait. In male flies where UAS-lbRNAi expression is under the control of the 1151GAL4 driver B″ the legs are held closer to the body and the leg-print is the consequence of a shuffling gait. In male flies where UAS-lbe expression is under the control of the 1151GAL4 driver the pattern of prints B′″ illustrates a bias towards one side, a consequence of the legs being abnormally positioned with respect to the body.

Both the RNAi-based attenuation of *lb* gene activity and the 1151-targeted over-expression of Lbe resulted in affected locomotion in surviving adult flies. This prompted us to test the performance of leg muscles with reduced and forced *lb* expression. Two different behavioural assays were performed. In the first one see Materials and Methods for technical details we evaluated the capacity of male flies to catch, maintain and rotate a small polystyrene ball Fig. 4A and Video S10–Video S12. All the wild type flies were able to catch and maintain the ball at least for 30 s, but some of the lbRNAi individuals failed to catch it and several of them were unable to maintain it compare Wt Video S10 with lbRNAi Video S11. In ball rotation assay the lbRNAi flies were also significantly less efficient Fig. 4A and Video S10, S11. The reduced muscle performance was consistent with morphological and ultrastructural alterations observed in leg muscles with attenuated *lb* function. Interestingly, the over-expression of Lbe also leads to dramatic defects in muscle performance. The majority of 1151>Lbe flies tested were unable to catch the ball, and only a few of them maintained it for a 30 s period with sporadic rotations Fig. 4A and Video S12. We speculate that the inability of 1151>Lbe flies to successfully perform the ball tests may be due to degeneration of dorsal tendons accompanying the altered leg muscle pattern.

To determine whether *lb* induced muscle defects influenced walking behaviour we performed a “leg print” test Fig. 4B. Adult flies walking on a carbon-coated slide leave tracks that can be used as a read-out of their gait. Wild type flies use an ‘alternating-tripod’ gait, characteristic of insects walking briskly. This gait leaves a pattern on the carbon-coated slide that is stereotypic and, easily recognised, and is shown in Fig 4B^i^. When *lb* levels are down- regulated using the UAS-lbRNAi under the control of the 1151-GAL4 driver, a very strong and noticeable difference is seen in the leg-prints Fig 4B^ii^. The legs can be deduced to be positioned closer to the body axis than in the wild type, and a shuffling-gait also results in a reduced stride-length. In contrast, the GOF phenotype that results from the use of the UAS-lbe under 1151-GAL4 control results in a pattern of leg prints, which reflect poor co-ordination and a spread-out positioning of the legs with respect to the body axis.

In some contexts, affects of muscle or tendon anatomy do not result in any obvious behavioural changes. In other situations, strong behavioural consequences are seen when sensitive components of the motor network are disrupted. Hence sensitive but readily performed behavioural assays are valuable in determining the contribution of specific components to normal motor function. In this case, given the expression of *lb* both in myoblasts and during morphogenesis, behavioural assays additionally allow the use of genetics to isolate interacting genes as well as alleles that may allow the dissection of the roles of *lb* between muscle specification and morphogenesis.

### Extrinsic Wingless signalling is required for *lbe* expression in leg myoblasts

**Figure 5:**
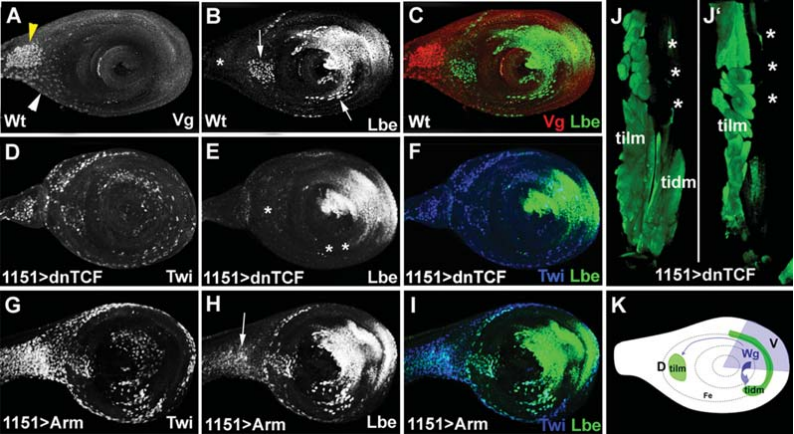
Wg signals are required for Lbe expression and play a key role in leg myogenesis A–I Confocal images of third instar leg imaginal discs. A–C Wild type leg disc stained for Lbe and Vestigial Vg. Lbe positive myoblasts are seen in leg disc proper B and green in merge C, while the myoblasts associated with the dorsal proximal region corresponding to the ventral thorax express Vg A and red in merge C. Vg myoblasts are devoid of Lbe asterisk in B. A Yellow arrowhead shows myoblasts expressing high levels of Vg and white arrowhead those expressing Vg at lower levels. D–F Leg disc, in which a dominant-negative TCF has been expressed in the myoblasts. Myoblasts are present as shown by anti-Twist antibody staining D and blue in merge F, but Lbe expression in myoblasts is lost asterisks in E and green in F. G–I Leg disc, in which activated Armadillo Arm transgene has been expressed in the myoblasts. Lbe is ectopically expressed in dorsal proximal myoblasts arrow in H and green in merge I, normally devoid of Lbe. Myoblasts are visualised with anti-Twi antibody G and blue in I. J–J′ show adult muscle phenotypes in the femur region induced by 1151-Gal4-driven forced expression of a dominant-negative TCF. Ventral muscles tidm are partially lost asterisks in J or completely lost asterisks in J′ and dorsal muscles tilm are severely affected. Muscles are visualised using MHC-tauGFP. Anterior views from the 3D reconstructions of confocal scans. K A schematic showing position of Lbe-expressing dorsal tilm and ventral tidm precursors of femur muscles green areas with respect to epithelial Wg expression domain violet triangle. The dorsal tilm myoblasts, located comparatively far from the Wg domain, receive a lower level of Wg morphogen long thin arrow than ventrally located tidm myoblasts short thick arrow.

Wingless signalling plays a pivotal role in specification of the proximal-distal and dorsal-ventral axes of the leg disc [15], [42]–[44], and has also been reported to control muscle development in the thoracic portion of the wing disc [25]. Also, in the embryo Wg promotes myogenic differentiation of mesodermal cells and is required for Lb expression in muscle and cardiac cells [36], [38]. We therefore wondered whether Wg regulated Lbe in the leg disc myoblasts. As stated above see also Fig. 5, asterisk, Lbe is excluded from the myoblasts populating the most proximal part of the leg disc, known to contribute to the ventral thorax. These myoblasts are located far from Wg-expressing ventral epithelium see schematic in Fig. 5K, suggesting that they do not receive enough Wg to express Lbe. Among proximal myoblasts the most distant with respect to the source of the Wg signal are myoblasts populating the dorsal-proximal area, the stalk. As shown in Fig. 5A yellow arrowhead and 5C, the stalk myoblasts express a high level of *Vestigial Vg,* which is also expressed at a lower level in some other proximal leg myoblasts arrowhead in Fig. 5A. To test whether Wg signalling regulates Lbe expression during appendicular myogenesis, we used 1151-GAL4 driver to over-express a dominant negative form of Wg effector dTCF or a constitutively active form of β-catenin/Armadillo. The attenuation of Wg signalling led to a dramatic fall in Lbe expression in myoblasts compare Figs 5B, C with 5E, F, whereas ectopic activation of the Wg pathway induced Lbe expression in proximal myoblasts associated with the stalk Fig. 5H, I, normally expressing Vg. Thus Wg signals from the ventral epithelium do indeed regulate Lbe expression in leg myoblasts and are most probably the origin of differential Lbe levels in ventral versus dorsal femur myoblasts Fig. 3E–G and scheme in Fig. 5K. The key role of Wg in leg myogenesis and differential requirements of ventrally and dorsally located myoblasts for Wg signals is supported by abnormal leg muscle pattern and preferential loss of ventrally located leg muscles in flies expressing a dominant negative form of dTCF Fig. 5J, J′.

Subdivision of leg disc associated myoblasts into Vg-positive proximal population and the Lbe-expressing myoblasts of the leg disc proper suggests that Vg and Lbe repress each other.

**Figure 6:**
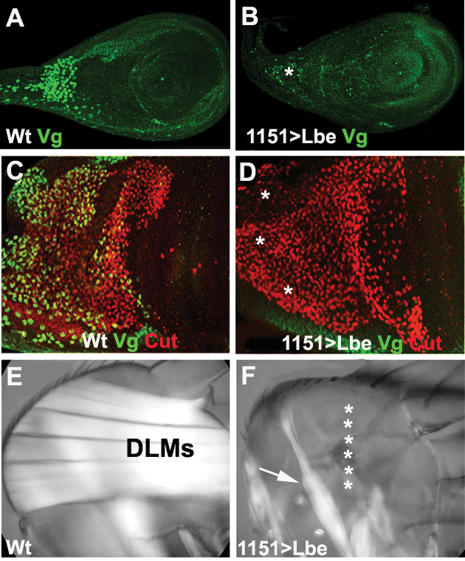
Ectopic Lbe represses Vg and interfere with flight muscle development A, B Third instar leg imaginal discs stained for Vg green. C, D Notum part of third instar wing imaginal discs stained for Vg green and for Cut red. In the wild type A, C Vg is expressed in a subset of leg disc myoblasts associated with the dorsal proximal region and in wing disc myoblasts known to give rise to thoracic flight muscles. In discs ectopically expressing Lbe in all myoblasts Vg is repressed asterisks in B and D. E, F Indirect flight muscles IFMs in the adult thorax viewed under polarised light. In the wild type hemithorax E, there are six DLMs and three sets of DVMs out of focus. F Ectopic expression of Lbe in myoblasts leads to disruption of IFMs; DLMs are lost asterisks while DVMs are severely reduced arrow.

When ectopically expressed Lbe was able to down-regulate Vg in proximal leg myoblasts as well as in wing disc associated myoblasts Fig. 6A–D. This resulted in disruption of thoracic IFM muscles Fig. 6E,F. Likewise in leg discs ectopically expressing Vg, Lbe was inhibited leading to an adversely affected leg muscle pattern Fig. S2. However, Lbe was not derepressed in stalk myoblasts of *Vg* null mutants and Vg was not expanded to myoblasts associated with leg disc proper after RNAi mediated attenuation of Lbe data not shown. This suggests that Lbe and Vg are not the only players that diversify leg disc myoblasts. As Lbe is activated in stalk myoblasts after overexpression of Arm Fig. 5H, I we speculate that Wg signalling contributes to this diversification process.

### FGF signalling promotes leg myoblast differentiation by inducing *lbe* and is involved in setting the number of myoblasts and resulting leg muscle fibres

**Figure 7:**
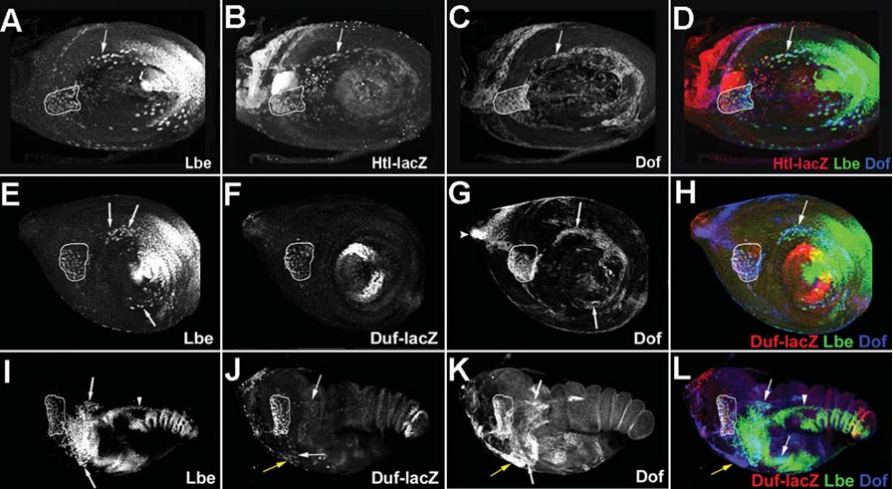
Components of FGF signalling pathway are expressed in all Lbe myoblasts A–L Confocal images of leg imaginal discs triple-stained with anti-Lbe A, E, I and green in merge D, H, L, anti-B-galactosidase against Htl lacZ B and red in merge D, anti-B-galactosidase against Duf –lacZ F, J and red in merge H, L, and anti-Dof antibodies C, G, K and blue in merge D, H, L. A–D A third instar leg disc showing coexpression of FGF receptor Heartless and the downstream target of Htl signalling, Dof in Lbe-positive myoblasts see outlined area and myoblasts indicated by arrows. E–L Progressive activation of Duf expression in Lbe myoblasts with active FGF signalling. In third instar leg discs E–H Duf is co-expressed with Lbe and Dof in dorsal femur myoblasts outlined area but not present in the ventral myoblasts arrows. At 3 hr-APF I–L in addition to dorsal myoblasts outlined area Duf is progressively activated in ventral Lbe- and Dof-positive leg myoblasts within the femur, tibia arrows in J and coxa yellow arrow in J segments. All leg disc myoblasts showing Lbe expression have active FGF signalling, shown by expression of Dof D, H, L. Arrowhead in L points to Lbe expressing long tendon.

It has been recently reported [33] that Htl-transduced FGF signalling plays an important role in the differentiation of abdominal and thoracic adult myoblasts, leading to the segregation of Duf-expressing founder cells. Here we show that the FGF receptor Htl and the read-out of FGF signalling, Dof, are coexpressed with Lbe in leg disc associated myoblasts Fig. 7. Lbe positive myoblasts with active Heartless signalling start expressing presumptive founder cell marker Duf-lacZ. At third instar, Duf-lacZ expression colocalises with Lbe in myoblasts of the dorsal femur muscle, tilm see ringed area in Fig. 7E–H suggesting a potential role of FGF in the differentiation of Twi-positive myoblasts into Lbe- and Duf-expressing cells. At the beginning of pupation, additional Lbe/Dof-positive myoblasts located in the tibia and coxa start to express low levels of Duf-lacZ arrows in Fig. 7I–L, indicating that activation of Lbe and Dof precedes that of Duf and represents an early event in myogenic differentiation.

**Figure 8:**
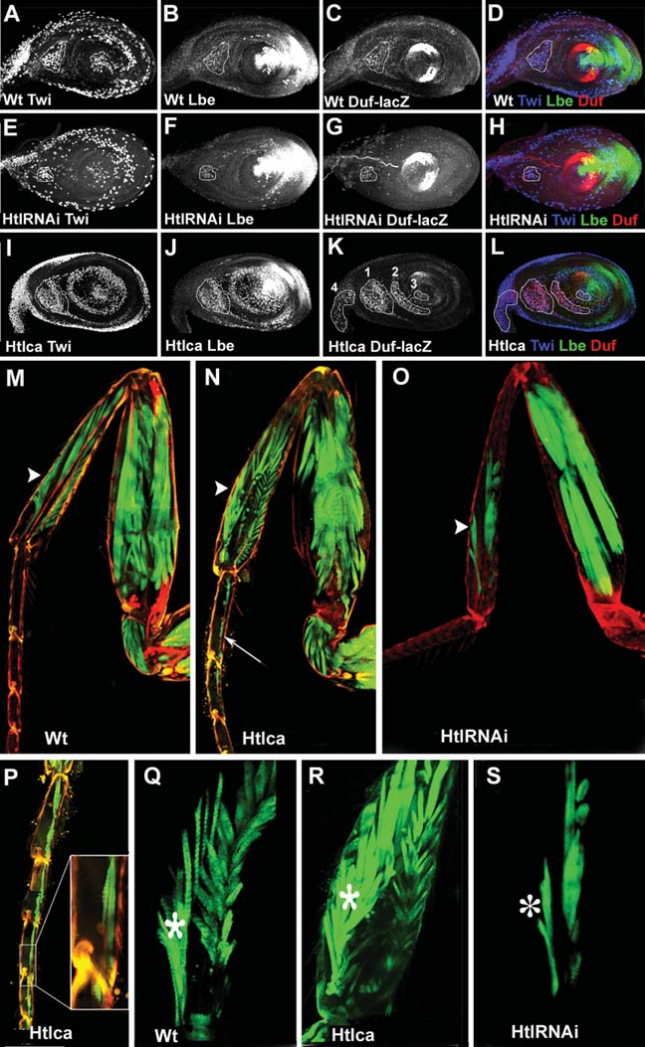
Htl-transduced FGF signals regulate number of leg myoblasts and resulting muscle fibres and when overexpressed promote expression of Lbe and Duf. A–L Confocal images of third instar leg discs stained for Twi, Lbe and Duf-lacZ. Leg disc A–D Control *duf* lacZ, and Leg disc E–H with attenuated Htl signalling *htl* RNAi, I–L with increased Htl signalling *htl*-ca, in the myoblasts. Attenuation of Htl expression leads to reduced number of myoblasts associated with the leg disc compare A and E. This is particularly obvious when comparing the number of Twi/Lbe/Duf expressing cells within the outlined area in the dorsal femur. In contrast, forced Htl expression leads to significantly increased number of leg myoblasts outlined area 1 in I–L. Moreover, Htl, when overexpressed, induces Lbe prematurely in all myoblasts in the leg disc proper and promotes differentiation of dorsally located myoblasts into Duf-positive cells K,L, outlined areas 2,3,4. Note that the only leg disc associated Duf-lacZ myoblasts that do not express Lbe are in area 4, corresponding to the proximal part of the leg disc that contributes to the ventral thorax. Surprisingly, Htlca also induces Lbe/Duf-lacZ expression in tarsal segments outlined area 3. These cells give rise to ectopic muscles in tarsal segments N,P otherwise devoid of muscles. M A wild type adult muscle pattern revealed in the leg expressing muscle-specific MHC-tauGFP green and tendon-specific 1151-DsRed red. Note that no muscles are detected in the tarsus. Arrowhead points to the dorsal tibia muscle, talm. N Gain of Htl signalling in leg myoblasts leads to the formation of supernumerary muscle fibres arrowhead in N. Ectopic muscles form in the tarsus arrow in N. O RNAi-mediated attenuation of Htl expression leads to the reduced number of muscle fibres arrowhead in O. P An enlarged view of tarsal segments shown in N. Ectopic muscle fibres align along the long tendon but most of them are not attached to the epithelium. Q An enlarged view of the distal tibia muscles in wild type R; in Htl gain-of-function and after RNAi-based Htl attenuation S The number of muscle fibres in the talm muscle indicated by asterisk is significantly increased in R and reduced in S.

To test the role of FGF in early steps of appendicular myogenesis and its influence on Lbe expression in leg myoblasts we analysed targeted attenuation or gain of function of the FGF receptor Htl using 1151-GAL4; MHC-tauGFP strain see Materials and Methods for details. Compared with the wild type Fig. 8A–D, the overall leg disc myoblast number appears reduced in HtlRNAi discs Fig. 8E–H. To precisely evaluate the effect of Htl on myoblast number we counted the Twi/Lbe/Duf-LacZ positive myoblasts in the dorsal femur tilm outlined area 1 in Figs. 8D, H, as being the first regionalised myoblast group to be differentiated. We found that in HtlRNAi third instar discs the number of tilm myoblasts 34,2; *n* = 5 was significantly reduced compared with the wild type 55,2; *n* = 5. In contrast, overexpression of the constitutively active form of the Htl receptor results in strongly increased number of leg disc associated myoblasts number of tilm myoblasts 118.5; *n* = 5 see also outlined area 1 in Fig. 8K and ectopic activation of Lbe and Duf in all leg segments Fig. 8I–L. Interestingly, in addition to premature activation of Lbe and Duf in the tibia, forced FGF expression leads to the formation of ectopic Lbe/Duf-positive myoblasts in the tarsus segments known to be devoid of muscles outlined area 3 in Fig. 8L. We also observe that in proximal stalk myoblasts, FGF induces Duf but not Lbe and that Duf activation but not Lbe activation is dorsally restricted Fig. 8J–L. Thus we hypothesise that Htl acts in cooperation with Wg to induce Lbe and with some dorsally expressed factors to activate Duf.

To gain a better understanding of the role of FGF in appendicular myogenesis we also investigated whether the Htl-induced alterations in the number of Lbe-expressing myoblasts and differentiating Duf-positive cells influenced muscle organisation in the adult leg Fig. 8M–S. Consistent with larval phenotypes, 1151-driven over-expression of Htlca led to the significantly higher number of fibres contributing to leg muscles arrowheads in Fig. 8M,N. For example, the average number of Htlca-induced muscle fibres counted in 10 adult legs of the tarsus levator muscle talm was 14.8 against 10 fibres in the wild type compare Fig. 8Q and 8R. However, the overall leg muscle pattern and capacity of fibres to interact with internal tendons appeared unchanged. To rule out any influence of Htl signalling on the specification and differentiation of tendon precursors in the leg, we used 1151Gal4;UASFP and SrGal4;UASGFP driver/sensor strains Fig. S1 and data not shown. We found no influence of Htl on specification or differentiation of tendon precursors in the leg Fig. S1 and data not shown, and no non-autonomous effect via tendons on myoblast number data not shown. In some legs after forced Htlca expression we observed enlarged internal tendons Fig. S1. This tendon alteration may be due to the increased number of muscle fibres that attach to the tendons. Thus FGF functions to set the number of myoblasts and resulting leg muscle fibres and appears to contribute to the activation of Lbe and differentiation of Duf-positive myoblasts. An instructive role of Htl in appendicular myogenesis is further supported by its capacity to induce formation of ectopic muscles in the tarsus arrow in Fig. 8N and Fig. 8P and by the reduced number of appendicular muscle fibres observed after targeted attenuation of Htl by RNAi Fig. 8O,S.

## Discussion

In both invertebrates and vertebrates, legs are locomotor appendages formed as an outgrowth of the body wall. Interestingly, the expression of the evolutionarily conserved *Distal-less Dll/Dlx* genes marks the initiation of appendage development in a broad spectrum of animal phyla [7], [9]. Our previous work revealed that *Drosophila* and vertebrate legs shared similar dorso-ventral distribution of multi-fibre muscle units [22]. Here we report evidence for common components of genetic circuitry that control both invertebrate and vertebrate leg muscle development. In particular we show that *lbe*, the *Drosophila* orthologue of the *Lbx1* gene, required for the migration and development of vertebrate limb muscle precursors [2], [3] is essential for establishing morphological, ultrastructural and functional properties of leg muscles in the fruit fly.

### *ladybird* - an evolutionarily conserved regulator of appendicular myogenesis

*ladybird/Lbx1* genes are known to determine individual cell fates of muscular and neural progenitors in *Drosophila* [36], [43] and in vertebrate embryos [2], [3]. The analysis of muscle phenotypes in mice lacking the functional *Lbx1* gene has led to the conclusion that *Lbx1* is involved in the interpretation of signals that guide appendicular muscle precursor migration [2], [3], [35]. In addition, analyses of *Lbx1* expression in different vertebrate species show it to be a hallmark of an appendicular myogenic programme involving delamination and migration of limb muscle precursors [1], [27]. Here we show that *ladybird* genes control leg muscle development in the fruit fly. This raises the intriguing possibility that appendicular myogenic functions of *ladybird/Lbx1* gene family were acquired early in evolution to generate a specialised type of appendage adapted for locomotion. Like *Lbx1* in vertebrates, *lb* marks an entry point in the leg muscle developmental programme and specifies properties of myoblasts that give rise to appendicular muscles. The most important feature of vertebrate appendicular myoblasts, which make them different from other myoblasts, is their capacity to undergo directed long-range migration. This feature is severely impaired in *Lbx1* knockout mice [2], [3]. In *Drosophila*, development of the leg muscles involves dorso-ventral myoblast positioning and subsequent movements during eversion of the leg discs, suggesting that *lb* can play a role in interpreting cues that direct these processes. Moreover, the leg-specific identity of muscle precursors is manifested by their capacity to interact with the internal tendons and form a unique pattern of multifibre muscles. The pattern and sarcomeric ultrastructure of muscle fibres are altered in flies with attenuated *lb* expression. Also, muscle-tendon junctions appear affected, suggesting that performance of *lb*-devoid muscles may be reduced. This possibility was fully confirmed by tests of muscle efficiency showing that *lb* activity is required for proper leg muscle function and thus for walking behaviour in the adult fly.

### *ladybird and* Wingless control diversification of leg disc associated myoblasts

The wing and leg imaginal discs contain the progenitors of both the appendages and the body wall of the thorax. Within the wing disc myoblasts underlay only the body wall epithelium, whereas the leg disc associated myoblasts are located on both territories. Our data demonstrate that Lbe is expressed exclusively in myoblasts that populate the leg disc proper and is excluded from the myoblasts underlaying proximal portion of the leg disc epithelium that contribute to the ventral thorax. This restriction depends on Wg signalling from the ventral epithelium that makes the most proximal myoblasts located far from Wg source incompetent to express Lbe. The key role of Wg in Lbe activation is confirmed by the extension of Lbe expression to proximal myoblasts in leg discs expressing a constitutively active form of Arm and by loss of Lbe in disc with attenuated Wg signalling. Interestingly, the Lbe-negative proximal leg disc myoblasts express *vg*, previously found to control IFM development [25]. When ectopically expressed *lbe* is able to repress *vg* and interfere with flight muscle development. In a similar manner, in gain-of-function conditions, *vg* represses *lbe*, leading to the formation of morphologically altered leg muscles. Thus we hypothesise that *lbe and vg* control two distinct programmes of muscle development and that *lbe* acts as an effector of Wg signalling in the diversification of leg disc associated myoblasts.

In addition, we observed that within the femur the level of *lbe* expression was significantly lower in dorsally than in ventrally located myoblasts. This is most probably due to a low Wg concentration in the dorsal region. Thus by differential regulation of Lbe, Wg appears to contribute to dorso-ventral diversity of muscle groups within the leg. This possibility is supported by the dorsally restricted muscle defects observed in *lb* gain of function mutants and by preferential loss of ventral muscles in the legs from flies over-expressing dnTCF.

### Heartless transduced FGF signals are required to set the number of leg myoblasts and promote their differentiation

It has been recently shown [28], [33] that the formation of the body wall muscles of the adult fly involves the Htl-directed differentiation of Duf-expressing cells. Here we present evidence that the FGF pathway plays an important role in setting the number and promoting the differentiation of myoblasts associated with the leg discs. We show that all the Lbe-positive myoblasts express FGF receptor Heartless and downstream intracellular protein Dof, known to be a specific read-out of FGF signalling [46]. We also demonstrate that forced expression of an activated form of FGF receptor Htl leads to an increased number of leg myoblasts and to their premature differentiation into Lbe-positive muscle precursors. Importantly, FGF signalling is also able to induce ectopic Duf expression in the dorsal region of all leg segments. As a consequence, we observe that a higher number of fibres contribute to the leg muscles. This indicates that regulated by FGF signals, the number of Duf-expressing myoblasts is directly related to the number of fibres composing adult leg muscles, consistent with a role of the FGF pathway in the differentiation of the adult abdominal muscle founders [33]. The instructive myogenic potential of the FGF pathway is also illustrated by the capacity of Htlca to induce myogenesis in tarsal segments otherwise devoid of muscle. The FGF-induced formation of tarsal muscles involves maintenance of Twist expression in tarsal adepithelial cells followed by differentiation of Lbe/Duf-positive myoblasts, thus indicating that activation of Lbe and Duf are obligatory steps in leg muscle development. Moreover, the observation that forced Htlca expression leads to the differentiation of supernumerary Duf-positive cells only in the dorsal portion of the leg disc suggests cooperation of FGF with a secreted signal from the dorsal epithelium. The observed inability of ectopic Htl to promote differentiation of the ventral population of myoblasts in the third instar larvae seems not to be maintained in later stages, since the number of fibres composing ventrally-located depressor muscles was also increased in legs from the 1151>Htlca flies. Thus we conclude that the FGF pathway promotes the differentiation of both dorsal and ventral myoblasts into Lbe/Duf-expressing cells, but that this process is delayed in the ventral region. The timely distinct differentiation of ventrally located myoblasts probably underlies a specific developmental programme enabling the formation of functionally distinct depressor muscles.

Taken together, our data provide evidence that in *Drosophila* like in vertebrates, multi-fibre appendicular muscles develop from a specialised pool of myoblasts expressing *ladybird/Lbx1* homeobox genes. *ladybird* genes determine leg-specific properties of myoblasts and muscle fibres, thus ensuring adaptation of the muscle system to walking behaviour. Given that Wg/Wnt and FGF signals promote leg muscle development in both *Drosophila* larvae and vertebrate embryos [26], [34], [47], we consider that further insight into *Drosophila* leg myogenesis should improve our understanding of the genetic control of appendicular muscle formation and function in general.

## Materials and Methods

### Drosophila strains

1151-Gal4 has been described elsewhere [25], [33]. 1151-Duf-lacZ and 1151-GFP are double transgenic strains generated from the 1151-Gal4 driver line combined with the Duf-lacZ line from A. Nose and UAS-GFPnls strain from Bloomington Stock Centre, respectively. To visualise adult leg muscles in different genetic contexts we generated the 1151-MHC-GFP muscle driver/sensor line by combining 1151-Gal4 with MHC-tauGFP strain a gift from E. Olson. The Gal4-UAS system [48] was used for directed expression of genes during adult myogenesis. To study the role of *lb* and investigate the role of Wg and FGF signalling in leg myogenesis, UAS-transgenic lines were crossed to 1151-MHC-GFP, 1151-GFP or 1151-Duf-lacZ. The UAS-*lbe* line has been described elsewhere [37] and UAS-*lb*RNAi lines were generated in the laboratory to investigate *lb* gene function tissue- and time-specifically. The UAS-*lb*RNAi^19/8^ line analysed here is a homozygous combination of UAS-*lbe*RNAi^19^ and UAS-*lbl*RNAi^8^ transgenic lines. It carries dsRNA producing pUAST constructs specifically targeting *lbe* and *lbl* genes. These constructs were generated by cloning, in inverted orientations, 800 bp cDNA fragments from the divergent 5′ regions of *lbe* and *lbl*. Heartless stocks Htl-lacZ a gift from A. Stathopoulous; UAS-Htlca and UAS-Htldn from the Bloomington Stock Centre; UAS-HtlRNAi obtained from K. VijayRhagavan's laboratory were used to study the role of FGF signalling. Stripe-Gal4; UAS-GFP line was obtained from G. Morata. UAS-dsRED strain was a gift from S. Heuser.

### Evaluation of RNAi-based attenuation of *lb* gene expression

To test the efficiency of UAS-lbRNAi constructs we crossed males from all generated lines with Actin-Gal4 virgin females. The resulting embryos were grown at 29°C for 7 h and then dechorionated and used for total protein preparation followed by Western blot. An equal number of embryos were used to obtain the all the analysed protein samples. Lb proteins were revealed using rabbit polyclonal anti-Lb antibody 1∶10000 detecting both Lbe and Lbl and mouse monoclonal anti-Lbe 1∶5000 [37]. To control protein loading a monoclonal anti-α-Tubulin antibody DSHB 1∶100 was used. Secondary anti-rabbit or anti-mouse antibodies 1∶5000 conjugated with peroxidase followed by the ECL chemiluminescence detection kit Amersham were used to reveal the Western blots.

### Dissections and mounting

Wandering third instar larvae were collected for larval dissections. White pre-pupae 0 h APF were collected and grown at appropriate temperatures for desired times before dissection. Crosses were set at 25°C and then either grown at 25°C or transferred to 29°C after 3 days of egg laying. Larvae and pupal preparations were dissected in phosphate-buffered saline PBS, fixed for 30 min in 4% paraformaldehyde PFA in PBS, washed and stained with appropriate antibodies. For leg dissections, adult legs were directly dissected in 4% PFA in PBS, fixed for 30 min, and washed twice for 10 min. All the preparations were mounted in 70% glycerol.

### Immunostaining and imaging analysis

The following primary antibodies were used: rabbit anti-Twi antibody, dilution 1∶200 generated in the laboratory; monoclonal anti-Ladybird early Lbe, dilution 1∶5000 generated in the laboratory; goat anti-LacZ, dilution 1∶1000 Biogenesis; rabbit anti-Dof, dilution 1∶500 a gift from M. Leptin; rabbit anti-Vestigial, dilution 1∶200 a gift from S. Carroll; and monoclonal anti-Cut, dilution 1∶1000 DSHB. The following secondary antibodies were used: donkey anti-rabbit and donkey anti-mouse Jackson antibodies conjugated to Alexa 488 or CY3 fluorochromes dilution 1∶300 and donkey anti-goat antibody conjugated to Biotin dilution 1∶2000 followed by Streptavidin-CY3 or -CY5 dilution 1∶300. All the preparations were visualised on ZEISS LSM 510 Meta or Olympus FV300 confocal microscopes. 3D reconstructions and image analyses were performed using Volocity^tm^ Improvision and Fluoview Olympus software.

### Electron microscopy

Transmission electron microscopy TEM material was fixed for 24 h in a modified Karnowsky's liquid 1% paraformaldehyde, 1% glutaraldehyde in 0.1 M phosphate buffer. The material was rinsed repeatedly in the same buffer and post-fixed for 2 h in 1% OsO_4_ in phosphate buffer, pH 7.4. After rinsing in 0.1 M phosphate buffer, the material was dehydrated in a graded alcohol series and acetone, and embedded in Epon 812 epoxy resin. The Epon blocks were cut on a Reihert Ultracut E ultramicrotome. Ultrathin sections were contrasted with uranyl acetate and lead citrate by the standard Reynolds method, and examined under a Zeiss EM 900 TEM at an accelerating potential of 80 kV.

### Muscle performance assessment

#### Ball test

Adult wild type, 1151>lbRNAi and 1151>lbe males were fixed on microscope slides in the “legs-up” position using a sealing solution prepared from double-sided 3M Scotch tape and N-heptane as a solvent. The polystyrene balls were prepared manually. Flies were tested for their ability to catch, maintain and rotate the ball. A Canon MVX4i video camera was used to record the ball test data.

#### Leg print test

Microscope slides were coated with soot using a candle flame. One- to two-day old flies were anaesthetised by chilling them on ice, their wings trimmed to prevent them from flying and on recovery placed on the carbon soot coated slide and allowed to walk. Tracks were photographed using a Canon digital SLR camera.

## Supporting Information

Figure S1 The 1151-GFP revealed internal leg tendons. A Wild type internal tendons of tibia and femur segments for tendon nomenclature refer to Soler et al., 2004; B Internal tendons in a lbRNAi leg. Note that all tendons are present, however the 1151-GFP labeling appears weaker in some of them eg. tadt yellow arrow. C The 1151-driven Lbe gain of function leads to abnormal pattern of the dorsal tilt tendon and reduced labeling of tadt and talt tendons yellow. This may result from the affected properties of muscle fibres that are unable to interact with their attachment sites leading to tendons degeneration. D Overexpression of Vg leads to dramatic alterations of internal leg tendons. Asterisks indicate lacking tendons. E Forced expression of HtlRNAi construct leads to a reduced tendon labeling, especially within the tibia segment. This is consistent with the loss of muscle fibres in the legs with attenuated Htl. C In legs expressing Htlca all tendons are present and some of them display a high levels of 1151GFP eg. tadt, red arrow.
6.86 MB TIF

Figure S2 Forced expression of Vg represses Lbe and affects leg muscle pattern. A, B Third instar leg discs stained for Lbe. B Myoblast-specific expression if Lbe is lost in leg discs ectopically expressing Vg asterisks. C Anterior and D posterior view of the MHC-tauGFP revealed femur muscles from legs overexpressing Vg. Arrows indicate abnormally patterned muscle fibres.
2.34 MB TIF

Video S1 Spatial distribution of Lbe expressing cells in a 0h APF leg disc double-stained for Lbe green and Twist blue.
5.24 MB MOV

Video S2 Spatial distribution of Lbe expressing cells in a 0h APF leg disc triple-stained for Lbe red and Twist blue and Stripe green.
8.05 MB MOV

Video S3 3D view of MHCtauGFP revealed wild type tibia muscles.
3.65 MB MOV

Video S4 3D view of tibia muscles from the adult lbRNAi flies mild phenotype.
2.51 MB MOV

Video S5 3D view of tibia muscles from the adult lbRNAi flies strong phenotype.
4.47 MB MOV

Video S6 3D view of MHCtauGFP revealed wild type femur muscles.
3.87 MB MOV

Video S7 3D view of femur muscles from the adult lbRNAi fly mild phenotype.
3.85 MB MOV

Video S8 3D view of femur muscles from the adult lbRNAi fly strong phenotype.
3.25 MB MOV

Video S9 3D view of femur muscles from the adult 1151>Lbe fly.
3.90 MB MOV

Video S10 The ball performance assay. A wild type male fly is shown.
7.63 MB MOV

Video S11 The ball performance assay. Effect of lb attenuation. A 1151>lbRNAi male fly is shown.
13.23 MB MOV

Video S12 The ball performance assay. Effect of gain of lb function. A 1151>lbe male fly is shown.
7.82 MB MOV
