# Supplementary figures and images for: Shaping Leg Muscles in Drosophila: Role of ladybird, a Conserved Regulator of Appendicular Myogenesis
Source: PLoS One. 2006 Dec 27;1(1):e122. doi: 10.1371/journal.pone.0000122 (PMC1762424; doi:10.1371/journal.pone.0000122)

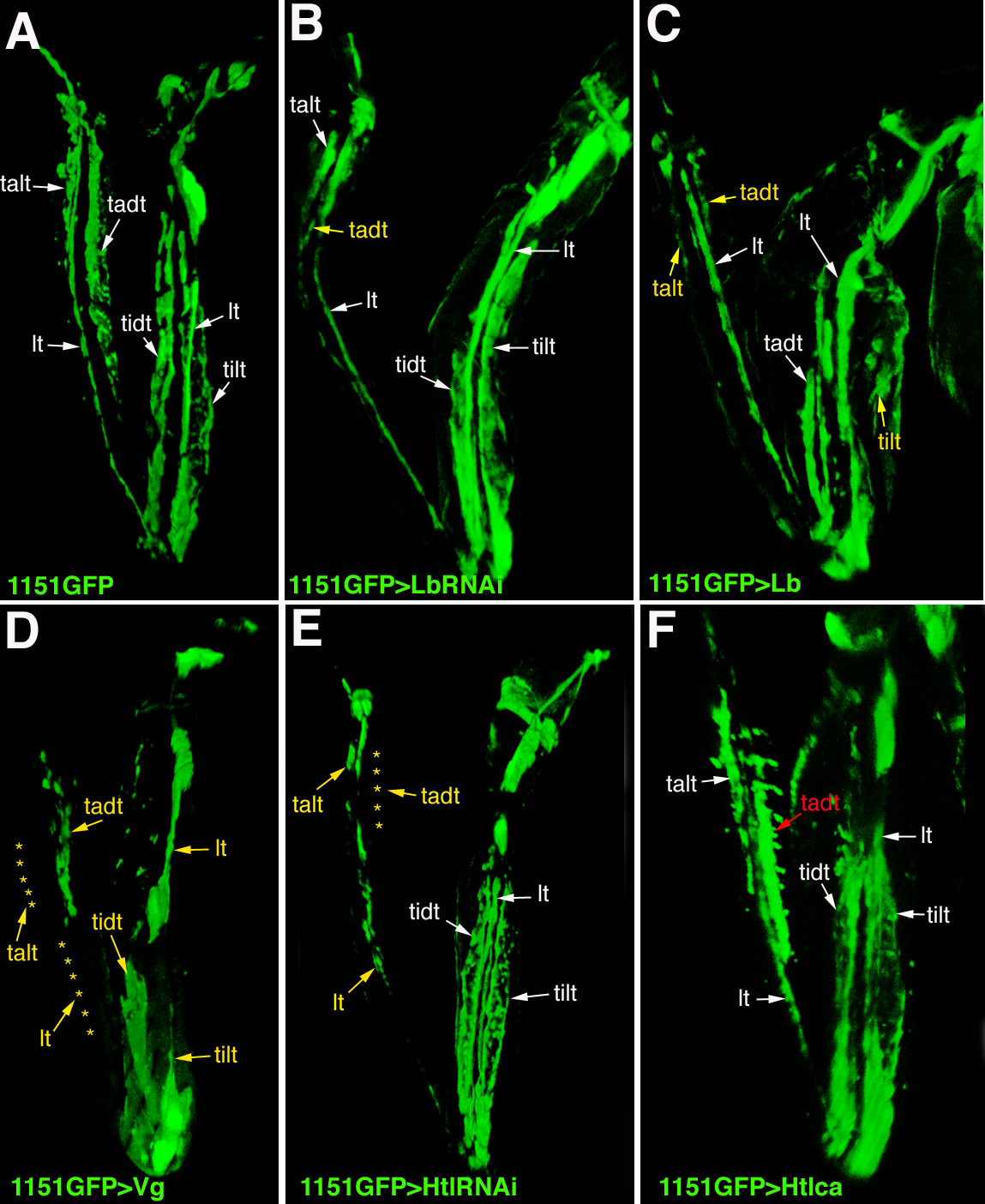

Supplement: Figure S1 — The 1151-GFP revealed internal leg tendons. A Wild type internal tendons of tibia and femur segments for tendon nomenclature refer to Soler et al., 2004; B Internal tendons in a lbRNAi leg. Note that all tendons are present, however the 1151-GFP labeling appears weaker in some of them eg. tadt yellow arrow. C The 1151-driven Lbe gain of function leads to abnormal pattern of the dorsal tilt tendon and reduced labeling of tadt and talt tendons yellow. This may result from the affected properties of muscle fibres that are unable to interact with their attachment sites leading to tendons degeneration. D Overexpression of Vg leads to dramatic alterations of internal leg tendons. Asterisks indicate lacking tendons. E Forced expression of HtlRNAi construct leads to a reduced tendon labeling, especially within the tibia segment. This is consistent with the loss of muscle fibres in the legs with attenuated Htl. C In legs expressing Htlca all tendons are present and some of them display a high levels of 1151GFP eg. tadt, red arrow. 6.86 MB TIF [file pone.0000122.s001.tif]

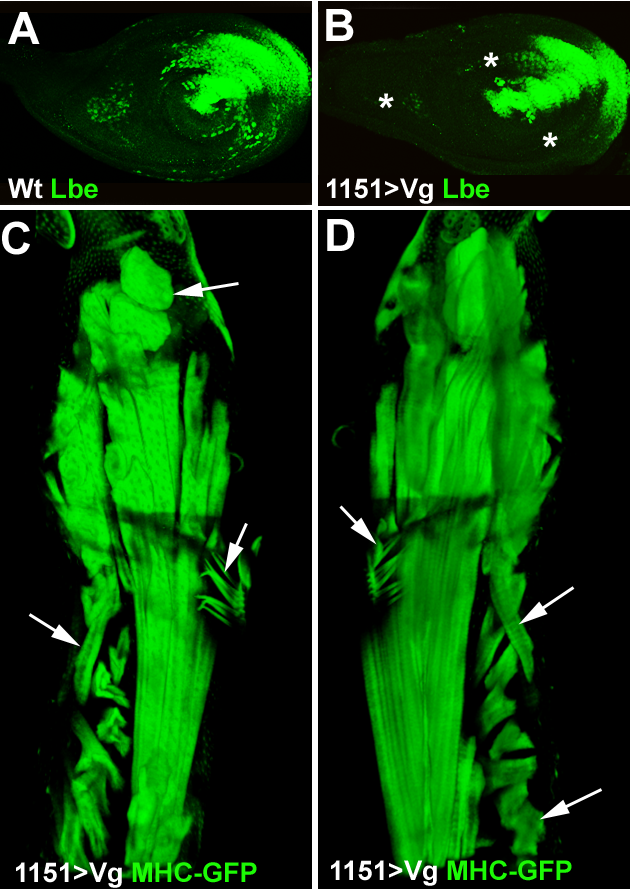

Supplement: Figure S2 — Forced expression of Vg represses Lbe and affects leg muscle pattern. A, B Third instar leg discs stained for Lbe. B Myoblast-specific expression if Lbe is lost in leg discs ectopically expressing Vg asterisks. C Anterior and D posterior view of the MHC-tauGFP revealed femur muscles from legs overexpressing Vg. Arrows indicate abnormally patterned muscle fibres. 2.34 MB TIF [file pone.0000122.s002.tif]
